# Body composition assessment by artificial intelligence can be a predictive tool for short-term postoperative complications in Hartmann’s reversals

**DOI:** 10.1186/s12893-024-02408-0

**Published:** 2024-04-15

**Authors:** Reshi Suthakaran, Ke Cao, Yasser Arafat, Josephine Yeung, Steven Chan, Mobin Master, Ian G. Faragher, Paul N. Baird, Justin M. C. Yeung

**Affiliations:** 1 Department of Colorectal Surgery, Western Health, Footscray, Melbourne, VIC 3011 Australia; 2 Department of Surgery, Western Precinct, University of Melbourne, Level 3, WCHRE, Sunshine Hospital, St Albans, Melbourne, VIC 3021 Australia; 3 Western Health Chronic Disease Alliance, Western Health, Footscray Hospital, Melbourne, VIC 3011 Australia; 4 Department of Radiology, Western Health, Melbourne, Australia

**Keywords:** Colorectal surgery, Sarcopenic obesity, Body composition

## Abstract

**Background:**

Hartmann's reversal, a complex elective surgery, reverses and closes the colostomy in individuals who previously underwent a Hartmann's procedure due to colonic pathology like cancer or diverticulitis. It demands careful planning and patient optimisation to help reduce postoperative complications. Preoperative evaluation of body composition has been useful in identifying patients at high risk of short-term postoperative outcomes following colorectal cancer surgery. We sought to explore the use of our in-house derived Artificial Intelligence (AI) algorithm to measure body composition within patients undergoing Hartmann’s reversal procedure in the prediction of short-term postoperative complications.

**Methods:**

A retrospective study of all patients who underwent Hartmann's reversal within a single tertiary referral centre (Western) in Melbourne, Australia and who had a preoperative Computerised Tomography (CT) scan performed. Body composition was measured using our previously validated AI algorithm for body segmentation developed by the Department of Surgery, Western Precinct, University of Melbourne. Sarcopenia in our study was defined as a skeletal muscle index (SMI), calculated as Skeletal Muscle Area (SMA) /height^2^ < 38.5 cm^2^/m^2^ in women and < 52.4 cm^2^/m^2^ in men.

**Results:**

Between 2010 and 2020, 47 patients (mean age 63.1 ± 12.3 years; male, *n* = 28 (59.6%) underwent body composition analysis. Twenty-one patients (44.7%) were sarcopenic, and 12 (25.5%) had evidence of sarcopenic obesity. The most common postoperative complication was surgical site infection (SSI) (*n* = 8, 17%). Sarcopenia (*n* = 7, 87.5%, *p* = 0.02) and sarcopenic obesity (*n* = 5, 62.5%, *p* = 0.02) were significantly associated with SSIs. The risks of developing an SSI were 8.7 times greater when sarcopenia was present.

**Conclusion:**

Sarcopenia and sarcopenic obesity were related to postoperative complications following Hartmann’s reversal. Body composition measured by a validated AI algorithm may be a beneficial tool for predicting short-term surgical outcomes for these patients.

**Supplementary Information:**

The online version contains supplementary material available at 10.1186/s12893-024-02408-0.

## Background

Hartmann’s procedure is a common emergency operation that leads to the formation of an end colostomy. Reversal of this stoma can be a complex operation, which requires considerable planning and patient optimisation, as it can be associated with considerable morbidity (mean 16.3%, range 3.6% – 50%) and mortality (mean 1%, range 0 – 7.1%) [1]. Common causes of morbidity include surgical site infections (SSIs) (5% – 30%), the development of an anastomotic leak (0% – 16%), as well as resulting in cardiopulmonary complications (1% – 14.6%); significantly impacting the length of stay, elevating healthcare costs and reducing the quality of life [1]. Unfortunately, there are no specific tools, medical algorithms, or validated scoring systems that can aid clinicians on how to predict outcomes in these patients when considering this complex operation.

Recently, body composition has gained attention in surgical practice, as it has been shown to help identify patients at risk of postoperative complications [2]. Several phenotypes have been described through body composition studies, utilising Lumbar 3 as the gold standard vertebral level of assessment [3]. Sarcopenia (low skeletal muscle index (SMI) and sarcopenic obesity (low SMI and high visceral adipose tissue (VAT)) have been associated with reduced oncological survival, in colorectal cancer, as well as increased postoperative complications, including surgical site infections and prolonged hospital stay [4]. This association may be due to compromised essential bodily functions (such as healing, immunity and strength) which contributes to a greater risk of infection and slower recovery.

Unfortunately, there are no studies within Australia or globally that have utilised body composition to predict short-term outcomes following Hartmann’s reversals. Our primary aim was to investigate the effects of sarcopenia and sarcopenic obesity in the prediction of short-term surgical outcomes following Hartmann’s reversals. We hypothesised that sarcopenic and sarcopenic obese patients would have increased short-term complications such as surgical site infections and worse overall morbidity.

## Method

### Study population

**Fig. 1:**
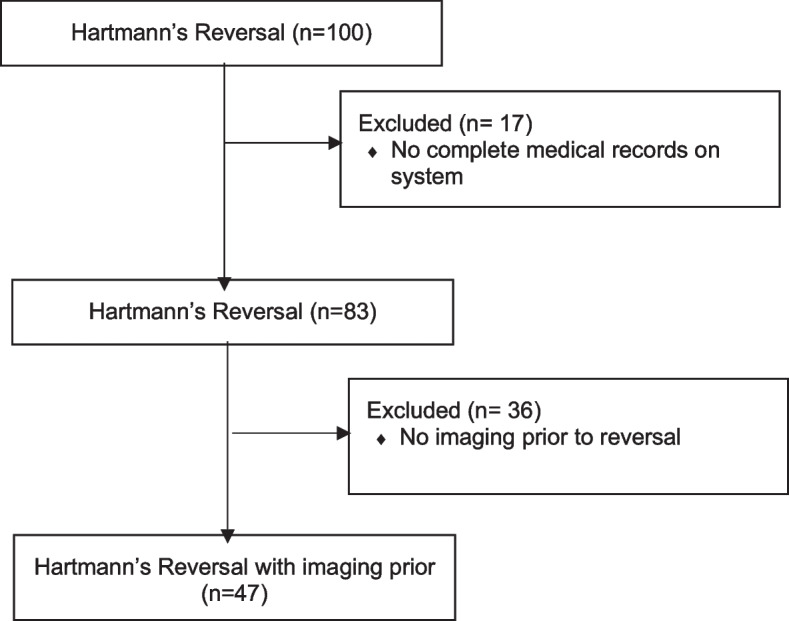
Flow diagram of patient inclusion /exclusion criteria available in the study

A retrospective study evaluating all patients who underwent Hartmann’s reversal at a tertiary referral metropolitan hospital, Western Health, in Melbourne, Australia, was carried out between January 2010 and January 2020. In our previous study, we identified all patients who had Hartmann’s operations using hospital coding (Medicare Benefits Schedule) and examined patients’ electronic medical records at our institution [5, 6]. Patients with incomplete data and those without a CT of the abdomen and pelvis prior to surgery were excluded – Fig. 1. Patient CT scans were identified on the hospital radiology software system, and DICOM (Digital Imaging and Communications in Medicine) images of the CT scans were downloaded for body composition segmentation and analysis.

### Body composition assessment and CT image analysis

Body composition derived from CT scan slices at the lumbar three vertebrae is considered the gold standard assessment for patients undergoing abdominal surgery [7, 8]. The two-dimension U-Net convolutional network AI algorithm was developed by our Department of Surgery, University of Melbourne AI research group (Parkville, Victoria) and validated by human researchers trained in body composition measurement and had a high validity score (an average dice coefficient of 0.98) using prior data in colorectal cancer patients (unpublished results). In addition, we checked our AI segmentation report with an expert manual reader (trained according to the Alberta protocol) using the dataset from this study. Random CT images were selected for manual segmentation and cross-checking (5 out of 47 CT images) using a semiautomated soft-ware (Slice-O-Matic version 5.0, Tomovision, Quebec,Canada). The average pixel accuracy for this comparison was 0.98 (0.98–0.99), and the average dice coefficient for all body composition segmentation was 0.98, with 0.97 for muscle, 0.97 for VAT and 0.98 for SAT.

### Data collection

Available patient data collected from electronic medical records included age, sex, body mass index (BMI), ASA class, comorbidities, medication use, alcohol intake and smoking status. Perioperative outcomes collected included the type of surgery (laparoscopic-assisted, open, need for defunctioning stoma), duration of surgery, and length of postoperative hospital stay. Postoperative complications collected included surgical site infections (superficial, deep, organ/space), anastomotic leak, bleeding, ileus, respiratory, cardiac, renal, and neurological disorders. We used the United States Centre for Disease Control and Prevention (CDC) to define surgical site infection (SSI) [9]. The incidence of SSIs was captured through documentation of clinical findings, wound cultures, CT imaging and further interventions. Special attention was placed during data collection for infection present at the time of the surgery (PATOS) to which subsequent SSI is attributed; none was found in our cohort of patients. Complications were classified using the Clavien-Dindo classification, and mortality was defined as death within 30 days after reversal [10].

### Definitions

Body composition variables measured included skeletal muscle surface area (SMA) (cm^2^), skeletal muscle radiodensity (HU), visceral adipose tissue surface area (VAT) (cm^2^), VAT radiodensity, subcutaneous adipose tissue surface area (SAT) (cm^2^) and SAT radiodensity (HU).

Sarcopenia in our study was based on the commonly derived skeletal muscle index (SMI), calculated as SMA/height^2^ [11]. SMI cut-off values of < 38.5 cm^2^/m^2^ in women and < 52.4 cm^2^/m^2^ in men, used previously to define sarcopenia in several European and North-American oncological studies at the level of the third lumbar vertebrae (L3) was utilised [11, 12]. In addition, skeletal muscle radiodensity (SMR), which has been used as a surrogate for muscle function, allows a more comprehensive assessment of sarcopenia [7, 13]. Body surface area (m^2^) was calculated using Mosteller’s formula [14].

In our assessment of fat composition within our patients, a VAT ≥ 100 cm^2^ was used to define visceral obesity, as per other studies in the literature [12, 15]. Gender-specific criteria for visceral obesity, such as VAT > 168 cm^2^ for men and > 80 cm^2^ for women, were considered; however, they are less commonly used within the literature and were not used in our study [12]. Finally, sarcopenic obesity, a new classification of a higher risk group of patients who are at a greater risk of cardiovascular complications and mortality, was defined as having a VAT ≥ 100 cm^2^ and SMI < 38.5 cm^2^/m^2^ in women and < 52.4 cm^2^/m^2^ in men respectively [12, 16].

### Statistical analysis

Statistical analyses were conducted using Stata/IC 16.0 software (StataCorp, 2020 College Station, TX, USA). The Shapiro–Wilk normality test was used to assess deviation from normally distributed data and reported as mean ± standard deviation (SD). Nonparametric data are reported as median (interquartile range). Student’s t-tests were used to compare the difference in distributions. Categorical variables were analysed using the χ^2^ or Fisher’s exact test. The threshold for statistical significance was *p* < 0.05.

## Results

One hundred patients had a Hartmann’s reversal, of which 53 were excluded, with 17 being due to the lack of medical record information, and 36 patients had no recent preoperative CT imaging available. The majority of the excluded cases were due to non-malignant causes (diverticular disease (*n* = 24, 67%) or due to iatrogenic injury (*n* = 4, 11%)). The absence of medical records may be linked to evolving health system documentation practices over a decade, while the lack of preoperative scans likely stems from a combination of surgeon preference and patient factors such as body habitus or clinical signs of parastomal hernias.Forty-seven patients had evidence of a CT abdomen and pelvis prior to their reversal of Hartmann’s procedure and were therefore included in our study. Of these, 22 (47%) had their initial Hartmann’s procedure for malignancy. The median time between CT imaging and the date of Hartmann’s reversal was 6 months (IQR 1.5 – 9.5 months). The mean age for our cohort was 63.1 ± 12.3 years and 28 (59.6%) were male. The median length of hospital postoperative stay was 6 days (IQR 5–12.5). The majority of the cases were performed open (*n* = 42, 89.3%), with the remainder being laparoscopic-assisted (*n* = 5, 10.6%). The median operating time was 249 (IQR 200–301) minutes. Patient characteristics are shown in Table 1. Surgical site infections were the most common postoperative complication; *n* = 8 (17.0%) (Table 2).

**Table 1:** Patient characteristics of those who underwent Hartmann’s reversal

*N* = 47	Sarcopenic obesity (*n* = 12)	No Sarcopenic obesity (*n* = 35)		n (%)
**Age (years)**	69.5 (SD 8.8)	61 (SD 12.6)	*p* = 0.05*	63.1 (SD 12.3)
**Gender**				
Female	3	16	*p* = 0.31	19 (40.4)
Male	9	19		28 (59.6)
**Smoking Status**				
Non-smoker	4	17	*p* = 0.43	21 (44.7)
Current Smoker	2	8		10 (21.3)
Ex-Smoker	6	10		16 (34.0)
**Diabetes**	1/12	7/28	*p* = 0.65	8 (17.0)
**Ischaemic Heart disease**	1/12	2/33	*p* = 1.00	3 (6.4)
**Respiratory disease**	1/12	9/35	*p* = 0.41	10 (21.3)
**Renal disease**	2/12	0/35	*p* = 0.06	2 (4.3)
**Previous malignancy**	0/12	1/35	*p* = 1.00	1 (2.1)
**Medications**				
Steroid use	1/12	2/33	*p* = 1.00	3 (6.4)
PPI use	3/12	7/28	*p* = 0.70	10 (21.3)
**Alcohol Use**	2/12	5/35	*p* = 1.00	7 (14.9)
**Weight (kg)**	83.4 (SD 13.0)	76.6 (SD 20.2)	*p* = 0.19	78.3 (SD 18.7)
**BSA (m**^**2**^**)**	2.00 (SD 0.18)	1.85 (SD 0.29)	*p* = 0.05*	1.89 (SD 0.28)
**BMI (kg/m**^**2**^**)**	27.4 (SD 3.2)	28.6 (SD 6.7)	*p* = 0.93	27 (IQR 23–33)
**Initial Indication for HP**				
** Large bowel obstruction**				26 (55.3)
** Malignancy**	6/12	16/35	*p* = 0.79	22
** Diverticular Stricture**	0/12	4/35	*p* = 0.56	4
** Perforated diverticular disease**	6/12	13/35	*p* = 0.51	18 (38.3)
** Iatrogenic**				3 (6.4)
**Colonoscopic perforation**	0/12	2/35	*p* = 1.00	2
**Colonic Ischemia**	0/12	1/35	*p* = 1.00	1

Fisher exact test used when appropriate

*PPI* Proton pump inhibitor, *BSA* Body Surface Area, *BMI* Body Mass Index, *HP* Hartmann’s Procedure, Diabetes – includes both type 1 and 2, and those on insulin and not on insulin. Respiratory disease – includes asthma, COPD, bronchiectasis, Renal disease – chronic kidney impairment

^*^Statistical significance

**Table 2:** Incidence of postoperative complications

**Complications**	**Sarcopenic obesity**	**No Sarcopenic obesity**		**Total number of patients with complications (%)**
SSI (superficial, deep, organ/space combined)	5/12	3/35	*p* = 0.02*	8 (17.0%)
Superficial SSI	5/12	2/12	*p* = 0.01*	7 (14.9%)
Deep SSI	3/12	0/5	*p* = 0.01*	3 (6.4%)
Organ/Space SSI	1/12	1/35	*p* = 0.45	2 (4.3%)
Loop ileostomy	1/12	4/35	*p* = 1.00	5 (10.6%)
Intraoperative injury	0/12	2/35	*p* = 1.00	2 (4.3%)
Postoperative bleeding	0/12	3/35	*p* = 0.56	3 (6.4%)
AKI	0/12	5/35	*p* = 0.31	5 (10.6%)
Incision hernia	2/12	1/35	*p* = 0.15	3 (6.4%)
VTE	0/12	1/35	*p* = 1.00	1 (2.1%)
Ileus	0/12	4/35	*p* = 0.56	4 (8.5%)
Small bowel obstruction	1/12	2/35	*p* = 1.00	3 (6.4%)
Anastomotic leak	0	0	n/a	0

Fisher exact test used when appropriate

*SSI* Surgical site infections, *AKI* Acute kidney injury, *VTE* Venous thromboembolism

^*^Statistical significance

Using our validated AI algorithm, we acquired values for skeletal muscle surface area (cm^2^) (SMA) and skeletal muscle radiodensity (HU) (SMR), visceral adipose tissue (VAT) area and radiodensity, and subcutaneous adipose tissue (SAT) area and radiodensity (Supporting Information). The prevalence of sarcopenia, visceral obesity and sarcopenic obesity are shown in Table 3. We found no significant differences in age, sex, prevalence of common co-morbidities, or even BMI between sarcopenic obese and non-sarcopenic obese patients (Table 1).

**Table 3:** Body composition types, prevalence and cut-offs for definitions at L3

Prevalence	n (%)	Reference for cut-offs
Sarcopenia definitions		
SMI: M < 52.4 cm^2^/m^2^ and F < 38.5 cm^2^/m^2^	21 (44.7%)	[11, 12]
SMA: M < 144.3 cm^2^ and F < 92.2 cm^2^	10 (21.3%)	[7, 13]
SMR: M < 38.5 HU and F < 34.3 HU	24 (51.0%)	[7, 13]
Visceral obesity definitions		
VAT > 100 cm^2^	31 (66.0%)	[12, 15]
VAT M > 168 cm^2^, F > 80 cm^2^	26 (55.3%)	[12, 17]
V/S > 0.4	41 (87.2%)	[15, 18]
Body Mass Index (BMI)		
Underweight (< 18.5 kg/m^2^)	0 (0%)	[19]
Healthy weight (18.5 – 24.9 kg/m^2^)	17 (36.1%)	[19]
Overweight (25.0 – 29.0 kg/m^2^)	11 (23.4%)	[19]
Obese (> 30 kg/m^2^)	19 (40.4%)	[19]
Sarcopenic obesity definition VAT > 100 cm^2^ + SMI: M < 52.4 cm^2^/m^2^ and F < 38.5 cm^2^/m^2^	12 (25.5%)	[12, 16]

*L3* Lumbar spine 3, *M* males, *F* females, *SMA* Skeletal muscle area, *SMI* Skeletal muscle index, *SMR* Skeletal muscle radiodensity, *VAT* Visceral adipose tissue, *SAT* Subcutaneous adipose tissue, *V/S* VAT/SAT, *BMI* Body mass index, *HU* Hounsfield units

There was evidence to demonstrate a significant association between all surgical site infections (SSIs) and sarcopenia (SMI < 52.4 cm^2^/m^2^ in males and < 38.5 cm^2^/m^2^ in females) (Table 4). A third (7/21) of sarcopenic patients were complicated by SSIs, which include superficial, deep, organ/space or a combination of the three. In comparison, only one (1/26, 3.8%) non-sarcopenic patient developed an SSI. There was a considerable difference in the proportion of SSIs amongst sarcopenic compared with non-sarcopenic patients (Fisher Exact Test (FET), *p* = 0.02); this finding persisted even after controlling for other variables such as age, gender, smoking status, diabetes, steroid use, ischaemic heart disease (IHD), renal disease and respiratory disease (logistic regression, *p* = 0.04). The relative risk of developing a surgical site infection was 8.7 times greater when sarcopenia was present. Similarly, there was also evidence to support an association between SSIs and sarcopenic obesity (FET, *p* = 0.02). Of the eight patients with SSIs, five (62.5%) had evidence of sarcopenic obesity. All surgical site infections were managed with a combination of oral or intravenous antibiotics. Those with deep SSIs required opening of the wound by the bedside, and one of the two with organ /space SSIs required radiologically guided drainage. No other body composition phenotype was associated with any other postoperative complications or length of stay within this study. Similarly, weight, body surface area (BSA) and body mass index (BMI) were not associated with length of stay or postoperative complications.

**Table 4:** Body composition and surgical site infections (SSIs)

	Combined SSIs		Superficial SSIs		Deep SSIs		Organ/Space SSIs	
Sarcopenia (SMI: M < 52.4 cm^2^/m^2^ and F < 38.5 cm^2^/m^2^)	7/21	***p* = 0.02**	6/21	***p* = 0.03**	3/21	*p* = 0.08	2/21	*p* = 0.19
No sarcopenia	1/26		1/26		0/26		0/26	
Visceral obesity (VAT > 100 cm^2^	6/31	*p* = 0.69	6/31	*p* = 0.40	3/31	*p* = 0.54	1/31	*p* = 0.63
No visceral obesity	2/16		1/16		0/16		1/16	
Sarcopenic Obesity	5/12	***p* = 0.02**	5/12	*p*** = 0.01**	3/12	***p* = 0.01**	1/12	*P* = 0.45
No sarcopenic obesity	3/35		2/35		0/35		1/35	

*SSI* Surgical site infection, *M* male, *F* female, *SMI* Skeletal muscle index, *VAT* Visceral adipose tissue

*p* < 0.05 as significant

## Discussion

Hartmann’s procedures are commonly performed for left-sided colonic pathology in the emergency setting. However, only a proportion of patients have their Hartmann’s reversed due to its complexity, poor patient functional status, and the associated incidence of postoperative complications. A previous study within this cohort of patients revealed a 25% of reversal rate [5]. Current methods of preoperative assessment have been limited in the identification of early postoperative outcomes [20]. Body composition has been shown to be useful in the prediction of early outcomes in patients having colorectal cancer surgery [2, 4, 15, 18, 21], but there is no current evidence to suggest that this can be used in the prediction of outcomes following Hartmann’s reversal (HR). There is evidence in the literature that patients with reduced muscle mass or density are associated with worse surgical outcomes [11, 21]. Targeted prehabilitation of patients with physiotherapy, exercise training and dietary optimisation has been shown to improve both short-term complications, including infection risk as well as long-term patient survival [22].

Our study identified that low skeletal muscle index (SMI) (sarcopenia) was associated with a greater incidence of surgical site infections (SSIs). Similar studies in colorectal cancer patients have found that sarcopenia was also associated with greater postoperative complications (17). Therefore, early identification of sarcopenic patients undergoing reversal who may benefit from prehabilitation programs may help to minimise postoperative complications and overall morbidity.

Almost a quarter of our patients (*n* = 12, 25.5%) undergoing Hartmann’s reversal had evidence of sarcopenic obesity. These patients were found to be older, and a significant proportion of them were also complicated by postoperative surgical site infections (*n* = 5, 62.5%). Other studies have similarly found that sarcopenic obesity was an independent risk factor for developing surgical site infections and was also associated with poor functional status and survival [11, 16].

The utility of a validated automated AI algorithm to identify these at-risk body composition phenotypes may allow clinicians to target appropriate therapy prior to placing at-risk patients towards Hartmann’s reversal surgery. We have shown that commonly measured body metrics such as weight, body mass index (BMI) or body surface area (BSA) failed to predict postoperative outcomes, highlighting the benefits of CT-based body composition measurement. Prehabilitation (tailored exercise, nutrition, and physiotherapy) has been shown to improve metabolic and physiological reserves within patients over a short period of time within the perioperative period. For example, studies have shown improvements in muscle function, even following a 4-week intervention which involved moderate-intensity aerobic and resistance exercise, dietary counselling, protein supplementation and anxiety reduction strategies [23]. Preoperative body composition assessments hold promise for tailoring prehabilitation programs, but further research is necessary to solidify these benefits and translate them into clinical practice.

Our study had several strengths and weaknesses. Only patients with pre-Hartmann’s reversal CT scans were included in this study, as reliance on historical imaging to measure body composition changes could be affected by factors during their emergency presentation. Although many studies do not report the time between imaging and operative management, our median time of 6 months could be comparatively longer than the 30-day and 3-month intervals reported in some studies [11, 12, 16]. Our AI algorithm was validated by human researchers trained in body composition measurement and had a high validity score, an average dice coefficient of 0.98. However, there is a current lack of international consensus on definitions for different body composition phenotypes and the thresholds used for their diagnosis were based on patient cohorts with different illnesses and were domicile outside of Australia. Other limitations of our study included the retrospective nature of our data, our limited sample size (due to the lack of CT scans in a third of patients) and the low incidence of postoperative complications; allowing adequate assessment of predominantly surgical site infections. In contrast to sarcopenia, where sex-specific cut-offs were employed, this study utilized a single threshold of visceral adipose tissue (VAT) ≥ 100 cm^2^ for the definition of visceral obesity. However, this approach aligns with the established consensus within the relevant literature. However, no Australian study in the literature currently exists for Hartmann’s reversals for comparison. We noted that our recruited patients for this study were those who had CT scans prior to surgery for two reasons. These were either patients who had colon cancer, and therefore the scans were performed as part of their cancer surveillance program or those who required complex planning for their Hartmann’s reversal, e.g. presence of a co-incidental abdominal ventral hernia.

## Conclusion

Sarcopenia and sarcopenic obesity are related to greater surgical site infections. Body composition analysis using preoperative CT scans may provide clinicians with an additional tool to stratify high-risk patients prior to Hartmann’s reversal, allowing these at-risk patients to have prehabilitation in order to improve outcomes.

### Supplementary Information

**Supplementary Material 1.**

## Data Availability

The datasets used and/or analysed during the current study are available from the corresponding author on reasonable request.

## Abbreviations

AI: Artificial Intelligence
AKI: Acute kidney injury
BMI: Body Mass Index
BSA: Body Surface Area
CT: Computed Tomography
DICOM: Digital Imaging and Communications in Medicine
HP: Hartmann’s Procedure
HU: Hounsfield units
L3: Lumbar spine 3
PPI: Proton pump inhibitor
SAT: Subcutaneous adipose tissue
SMA: Skeletal muscle area
SMI: Skeletal muscle index
SMR: Skeletal muscle radiodensity
SSI: Surgical site infections
VAT: Visceral adipose tissue
V/S: VAT/SAT
VTE: Venous thromboembolism
