# Supplementary material for: Body composition assessment by artificial intelligence can be a predictive tool for short-term postoperative complications in Hartmann’s reversals
Source: BMC Surg. 2024 Apr 15;24:111. doi: 10.1186/s12893-024-02408-0 (PMC11017666; doi:10.1186/s12893-024-02408-0)
Supplement: Supplementary file 1 — Supplementary Material 1. [file 12893_2024_2408_MOESM1_ESM.docx]

**Supporting Information 1**

**Body composition on computed tomography (CT) level L3 imaging prior to Hartmann’s Reversal**

| **Body composition variables** | **mean ± SD** |
| --- | --- |
| Skeletal muscle area (cm^2^) | 137.89 ± 34.57 |
| Muscle radiodensity (HU) | 36.25 ± 7.47 |
| VAT surface area (cm^2^) | 152.04 ± 102.37 |
| VAT radiodensity (HU) | -91.07 ± 34.94 |
| SAT surface area (cm^2^) | 196.49 ± 102.7 |
| SAT radiodensity (HU) | -96.82 ± 11.83 |

VAT – visceral adipose tissue, SAT – subcutaneous adipose tissue, HU – Hounsfield units
